# Assessment of Collaborative Robot (Cobot)-Assisted Histotripsy for Venous Clot Ablation

**DOI:** 10.1109/TBME.2020.3023630

**Published:** 2021-03-18

**Authors:** Kenneth B. Bader, Samuel A. Hendley, Viktor Bollen

**Affiliations:** University of Chicago, Chicago, IL 60637 USA; University of Chicago.

**Keywords:** Collaborative Robot, Histotripsy, Image-Guided Therapy

## Abstract

**Objective:**

The application of bubble-based ablation with the focus ultrasound therapy histotripsy is gaining traction for the treatment of venous thrombosis, among other pathologies. For extensive clot burden, the histotripsy source must be translated to ensure uniform bubble activity throughout the vascular obstruction. The purpose of this study was to evaluate the targeting accuracy of a histotripsy system comprised of a focused source, ultrasound image guidance, and a collaborative robot (cobot) positioner. The system was designed with a primary emphasis for treating deep vein thrombosis.

**Methods:**

Studies to test treatment planning and targeting bubble activity with the histotripsy-cobot system were conducted in an *in vitro* clot model. A tissue-mimicking phantom was also targeted with the system, and the predicted and actual areas of liquefaction were compared to gauge the spatial accuracy of ablation.

**Results:**

The system provided submillimeter accuracy for both tracking along an intended path (within 0.6 mm of a model vessel) and targeting bubble activity within the venous clot model (0.7 mm from the center of the clot). Good correlation was observed between the planned and actual liquefaction locations in the tissue phantom, with an average Dice similarity coefficient of 77.8%, and average Hausdorff distance of 1.6 mm.

**Conclusion:**

Cobots provide an effective means to apply histotripsy pulses over a treatment volume, with the ablation precision contingent on the quality of image guidance.

**Significance:**

Overall, these results demonstrate cobots can be used to guide histotripsy ablation for targets that extend beyond the natural focus of the transducer.

## I. INTRODUCTION

HISTOTRIPSY is an ablative form of focused ultrasound under development for the treatment of thrombo-occlusive disease [1], [2], among other clinical targets [3]. Ablation occurs not as a direct result of the ultrasound energy, but due to the mechanical activity of a bubble cloud that imparts lethal strain to the tissue [4]. In order to achieve sufficient tension to nucleate bubble clouds, histotripsy transducers are highly focused [5] and bubble activity is restricted in close proximity to the focal zone [6]. Venous obstructions can extend several centimeters along the length of the vessel, and be one centimeter or more in diameter [7], [8]. To ensure uniform bubble activity throughout the entire obstructed vessel, the transducer must be translated. With imaging feedback to ensure sufficient bubble activity for thrombus ablation throughout the occlusion, recanalization of the target vessel could be achieved using a single translational sequence (i.e. only a single pass of histotripsy exposure would be necessary along the vessel) [9].

Robotic systems are one potential means to facilitate the application of histotripsy activity over an extended volume. Collaborative robotic systems, or cobots [10], were developed in response to the need for easy automation, and can be operated without advanced programming knowledge. Integrated sensors enable cobots to be used in close proximity with humans, unlike industrial robotic systems [11]. When coupled with image guidance, a cobot-assisted histotripsy system will facilitate the planning and execution of treatment for venous thrombosis. Cobots have six degrees-of-freedom, which enable arbitrary angulation of the transducer relative to the target [12], [13]. The cobot joints can be adjusted manually to enable coarse position of the transducer over a range of nearly a meter. Positioning the cobot with submillimeter precision can also be achieved via direct commands to the system. These features represent a significant improvement in the usability of cobots compared to the three-axis robots systems employed previously for the delivery of therapeutic ultrasound [12], [13], [14]. Cobots have been integrated with ultrasound imaging systems [15], [16], and may provide interventionalists with a flexible tool to execute extensive histotripsy treatment paths in future clinical histotripsy systems.

In this study, we examine a cobot-histotripsy system for the treatment of venous thrombosis ablation *in vitro* [1], [17], [18]. The system was tested in its capacity for: 1. Mechanical repeatability, and the functionality of a graphical user interface for manual placement of waypoints under image guidance, 2. Targeting bubble cloud activity along the length of a clot, and 3. Spatial accuracy of ablation. Mechanical repeatability and bubble cloud targeting were assessed in a flow channel containing a human whole blood clot. This experimental design mimics venous occlusion, but does not enable precise co-registration of the *in situ* imaging used to plan the locations of histotripsy exposure with *post hoc* assessment of the ablation zone. Therefore, the spatial accuracy of ablation was tested in a tissue mimicking phantom embedded with fiducials. The Dice similarity coefficient and Hausdorff distance were used to quantitatively compare the predicted and actual ablation locations.

## II. METHODS

### A. System

**Fig. 1.**
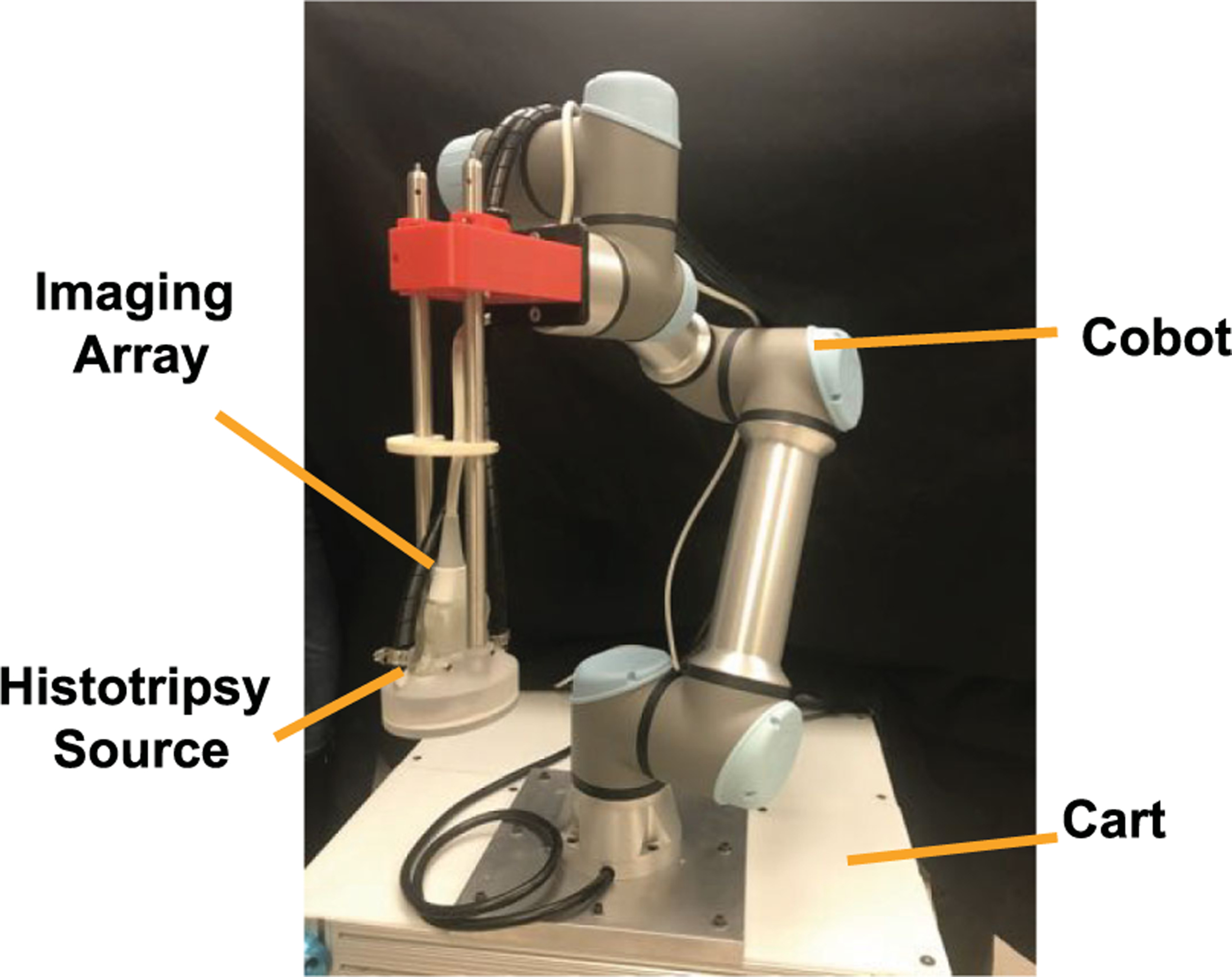
Image of histotripsy-cobot system. The histotripsy source was translated with a collaborative robot (cobot UR5e, Universal Robotics, Odesnse, Denmark). A confocal ultrasound imaging array was used to guide for the application of histotripsy pulses. The entire system was mounted on a cart to facilitate transport. Programming for the system was achieved via a Graphical User Interface (GUI).

An image of the experimental histotripsy system is shown in Fig. 1. A transducer prototyped for the ablation of thrombi in the femoral vein was used to generate bubble activity [19]. The source had an elliptic geometry (major and minor axes of 9 and 7 cm, respectively, and 6 cm focal length) with eight sector elements which operated at a fundamental frequency of 1.5 MHz. When driven under linear pressure conditions (e.g. low amplitude electrical excitation), a single elliptically shaped focal volume was generated with −6 dB widths of 1.1 × 0.9 × 4.8 mm. All elements of the source were driven in parallel by a custom build class D amplifier system [20]. A coaxial diagnostic ultrasound array (L11-5v, fundamental frequency 7.8 MHz, Verasonics, Inc, Kirkland, WA, USA) placed through a central window in the transducer was used for image guidance. The imaging array was controlled by a research ultrasound platform (Vantage 128, Verasonics, Inc, Kirkland, WA, USA).

A six degree-of-freedom cobot was used to translate the histotripsy system (UR5e, Universal Robotics, Odense, Denmark). The cobot was capable of carrying a 5 kg payload with ± 0.03 mm accuracy over its 850 mm reach, well within the width of a typical catherization bed [21]. Each axis of the cobot supported ± 360° rotation at an upper speed limit of 180°/s (approximately 1 m/s translational speed of the transducer). The entire system was mounted on a mobile cart equipped with stabilizing legs that were deployed during histotripsy insonation (cart dimensions 71.1 × 121.9 × 94.1 cm).

A custom graphical user interface (GUI) was developed in MATLAB (Mathworks, Natick, MA, USA) to control the cobot. When activated in freedrive mode by the GUI, the cobot acted as a zero-gravity support to enable manual positioning of the transducer. Fine positioning of the transducer could be achieved via the digital joystick interface on the GUI. To set the treatment path, waypoints (e.g. axes positions for the cobot that coincide with containment of the histotripsy focal zone within the clot) could be recorded on the GUI. A path through the clot was determined using an interpolation algorithm along the waypoints.

### B. Mechanical Repeatability

The objective of the first study was to test the accuracy of the system for tracking a target vessel based on user placement of waypoints under image guidance. This study serves as a measure of the system precision in pre-treatment planning, and as a baseline for the mechanical repeatability of the system and GUI under user guidance. Waypoints were set along a 1-cm length of a model vessel with uniform cross section (6.35-mm inner diameter and 0.79-mm wall thickness latex tubing, McMaster-Carr, Elmhurtst, IL, USA) submerged in an acrylic tank (dimensions 36 × 36 × 30 cm) of degassed (20% dissolved oxygen), filtered (10 *μ*m pore size), reverse osmosis water. The water was heated to physiologic temperature (37.3 ± 0.5°C) using a custom-built temperature controller circuit (ITC-308, Inkbird, Pengji Industrial Zone, Luohu District, Shenzhen, China) and submerged heating elements (HT 300 Titanium, Won Brothers, Fredericksburg, VA, USA).

**Fig. 2.**
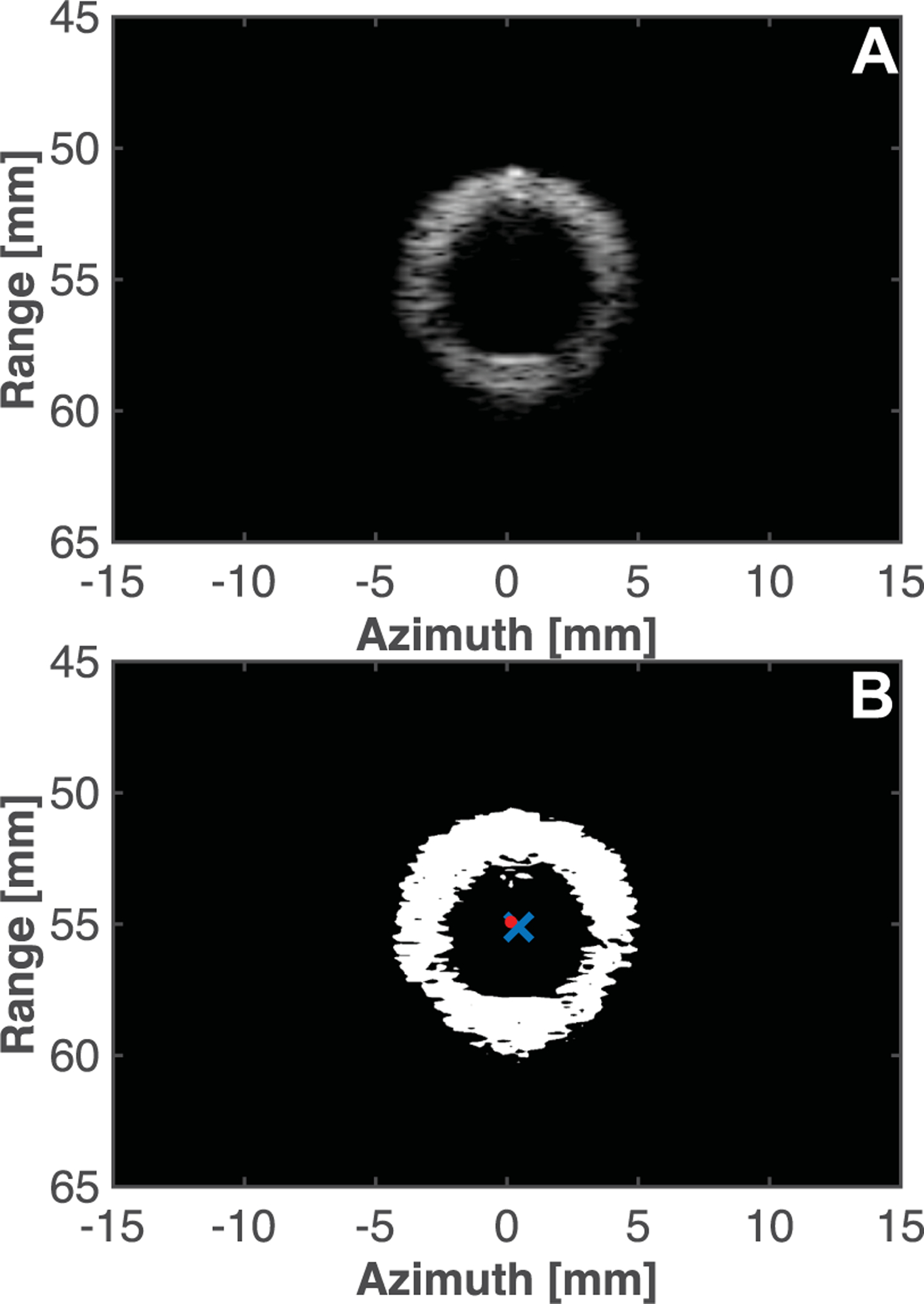
(A) B-mode image of the lumen cross section acquired with the imaging array confocal with histotripsy source. (B) B-mode processed with Otsu’s method. The red dot denotes the histotripsy focus, and the blue cross denotes the centroid of the lumen. The histotripsy focal location in the imaging plane was determined *a priori* by generating a bubble cloud in degassed water, and visualization with B-mode imaging. The center of the bubble cloud was marked with a cursor in the imaging window. This position was stored and compared to centroid of the lumen. In this example, the distance between the target location (centroid of the lumen, blue cross) and the actual placement of the histotripsy source (histotripsy focus, red dot) was 0.35 mm.

Prior to planning the path of the histotripsy system, the transducer focus was located by generating a bubble cloud in degassed water outside the lumen. The bubble cloud was visualized with B-mode imaging. A cursor on the imaging window was used to mark and save the center position of the bubble cloud. The position of the focus was also stored by the GUI. The histotripsy source/imaging array were aligned such that that the lumen cross section was visible in the image, and the histotripsy focus was placed in the center of the model vessel. For reference, the range resolution of the 7.8 MHz imaging sequence was approximately 0.4 mm [22]. The system was then scanned along the length of the model vessel (i.e. along the elevational plane of the imaging array), setting waypoints approximately every 5 mm to contain the transducer focus within the center of the lumen. The interpolation algorithm set a 1 cm path along the length of the lumen, stopping in 0.5 mm increments (approximately 20 locations in total). The one centimeter extent explored here is similar to other pre-clinical histotripsy clot ablation studies [1], [14], [23], [24], but would be several centimeters shorter than the observed burden for venous thrombosis patients [7]. At each location, a B-mode image was acquired of the lumen and downloaded for processing offline. The grayscale pixel values in the B-mode image associated with the lumen were determined using Otsu’s method in MATLAB (Fig. 2). The distance between the centroid of lumen and the histotripsy focus was used as a metric of accuracy for treatment planning. That is, studies here provide assessment of operator accuracy in the placement of waypoints along a model vessel under the image guidance system, and the cobot to track along those waypoints.

### C. Accuracy of Histotripsy-Clot Targeting

Human whole-blood clots were manufactured following an established internal review board (IRB) review-approved protocol [1]. Venous blood was drawn from a volunteer patient undergoing invasive catheterization procedures at the University of Chicago Medicine cardiac catheterization laboratory. Blood aliquots of 2 mL were transferred into borosilicate glass Pasteur pipettes (Fisher Scientific, Pittsburg, PA, USA). Clotting occurred as the blood aliquots were incubated in a water bath at 37 °C for 3 hrs. The formed clots were stored at 4 °C for a minimum of 3 days to allow for full retraction [25], and used within a two weeks period.

Prior to histotripsy exposure, the clot was cut to 1 cm in length and inserted into the lumen of the model vessel (latex tubing described in previous section) perfused with human fresh frozen plasma (0.65 mL/min flow rate [1], [26]). Waypoints were assigned via the GUI along the length of the clot using brief histotripsy pulses (less than one second total exposure). At each waypoint, the transducer was positioned was adjusted such that bubble activity was contained within the clot [23]. Three waypoints were designated for each clot (approximately 5 mm separation). Once waypoints were assigned, the GUI interpolation algorithm generated an automated path for the histotripsy source to translate along the length of the clot in 0.5 mm increments. At each location, 500 histotripsy pulses of 3.33 *μ*s duration (five cycles duration of the 1.5 MHz fundamental frequency) and 35 MPa peak negative pressure were applied at a 40 Hz rate. Based on previous studies, these insonation conditions were chosen to produce strong clot ablation while minimizing pre-focal bubble cloud activity [1], [14].

The locations of intense bubble cloud activity generated by the histotripsy pulses were assessed via passive cavitation imaging [27]. Passive cavitation imaging is significantly more sensitive to the detection of bubble activity [28] and a better predictor of the ablation extent along the azimuthal image dimension compared to active imaging techniques [9]. These advantages of bubble detection with passive cavitation imaging come at the cost of a reduced range resolution relative to active imaging methods [27], [29]. During the histotripsy excitation, the imaging array was used to record bubble cloud-induced acoustic emissions. Emissions were processed offline with the robust Capon beamformer to assign the acoustic power (*P*_*RCB*_) generated by histotripsy bubble activity as a function of location in the imaging plane $\vec{r}$ [30]:

(1) $$P_{\text{RCB }}(\vec{r})=\frac{4\;\pi d(\vec{r})}{T_{H}\rho c}\frac{1}{\hat{a}(\vec{r})UV{\left(\frac{1}{\lambda^{2}}+\frac{2V}{\lambda }+V\right)}^{-1}U^{T}\hat{a}(\vec{r})}$$

where *d* is the mean distance between the pixel location and the position of the elements of the array, and *ρ* and *c* are the medium density and sound speed, respectively. The vector ***â*** is the data-adaptive steering vector solved via the Lagrange multiplier *λ*, and ***U*** and ***V*** are matrix solutions to the eigenvalue decomposition of the correlation matrix $\int_{0}^{T_{H}}s_{n}(t)s_{m}(t)dt={\text{UVU}}^{\text{T}}$. Here, *s*_*n*_ is the steered signal received by the *n*th element, and *T*_*H*_ is the duration of the received signal. Due to data transfer rate limitations, passive cavitation images were acquired once every tenth histotripsy pulse (50 total passive cavitation images per insonation location, ~1000 images per clot).

**Fig. 3.**
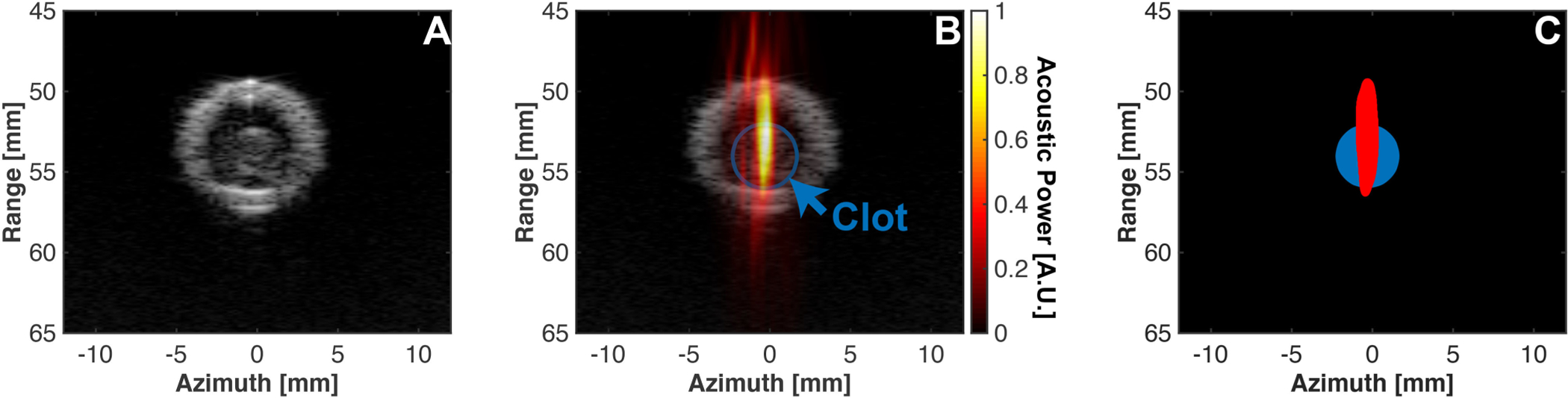
(A) B-mode image of clot in the lumen acquired prior to the application of histotripsy pulses. (B) Overlay of passive cavitation image (hot color map) with B-mode image (grayscale image). Note the strong emissions within the clot. (C) Binary thresholding of clot (blue area) and cavitation emissions within 1 dB relative to the maximum acoustic power (red area).

Prior to the application of histotripsy pulses, a B-mode image was acquired at each insonation location and segmented manually to determine the position of the clot (Fig. 3). At each insonation location in the clot, all 50 passive cavitation images were averaged pixel-wise, and the centroid of emissions (e.g. the location of strongest bubble activity) was computed [29]. The distance between the centroid of the passive cavitation image and the center of the clot was tabulated as a metric of bubble targeting accuracy for the system.

The Dice similarity coefficient (*DSC*) was computed to assess the degree of histotripsy energy contained within the clot or within the lumen [31]:

(2) DSC=2(A∩B)/(A+B)

where *A* is the area of bubble cloud emissions within 1 dB of the peak emission level, and *B* is the area of the clot or lumen. The clot mass was measured before and after histotripsy exposure using a digital balance. To remove excess fluid, the clot was blotted [24]. The thrombolytic efficacy was reported as the percent mass loss of the clot.

### D. Spatial Accuracy of Ablation

To test the ability of the system to provide good spatial accuracy of ablation, bubble activity was generated in a phantom established for the development of histotripsy technology [32], [33]. Briefly, CPD porcine blood was obtained from a commercial vendor (Lampire Biological, Pipersville, PA, USA), and centrifuged for 10 min. Red blood cells were separated from the plasma and buffy coat supernatant. Agarose powder (4.0 g, 1% by volume) and sodium chloride (3.6 g, 0.9% by volume, Sigma-Aldrich, St. Louis, MO, USA) were dissolved into 400 mL of ASTM Type I water (18 MOhm-cm resistivity) by heating in a microwave (700 W power) in 30s increments until clear. The agarose mixture was transferred to an ultrasonic cleaning bath heated to 65 °C while continuously evacuating (~2 kPa) for 30 min. Following degassing, the liquid agarose was poured to fill approximately one third of a rectangular acrylic mold (~5 cm × 5 cm × 5 cm) and allowed to solidify. Between the layers of solidified agarose, a 5% v/v red blood cell/agarose mixture was pipetted onto the solidified agarose slab to form a layer of approximately 500 *μ*m thickness. Liquefaction zones generated in the phantom have a similar morphology to ablation zones generated in *ex vivo* tissue [32].

**Fig. 4.**
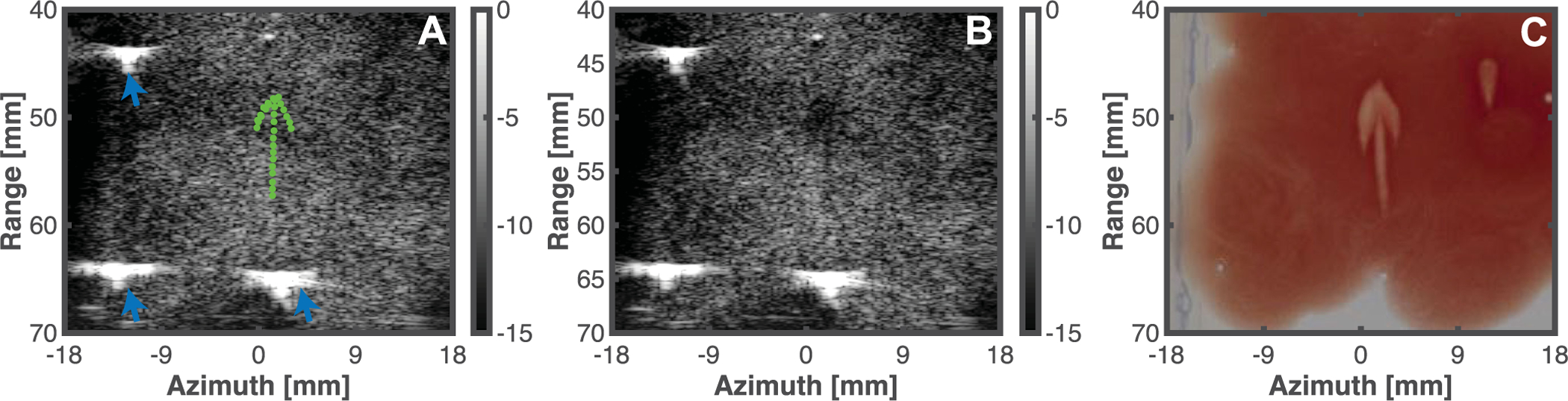
(A) *A priori* B-mode image of the red blood cell phantom. The green points indication locations that the cobot will translate the histotripsy focus to within the phantom, set via the GUI interface. Fiducials embedded in the phantom are noted with blue arrows. (B) *Post hoc* B-mode image of the phantom following histotripsy exposure. (C) Gross visualization of the phantom following histotripsy exposure. The grayscale colormap in panels A and B are Decibels relative to the maximum pixel value.

Phantoms were placed in the tank of degassed water described in Section II-C. The imaging plane of the confocal array was aligned with the red blood cell layer using the cobot in freedrive mode. Brief histotripsy pulses (less than one second total exposure) were used to confirm the focal zone location in the imaging plane for insonation of the phantom. A cursor was used to draw an area or path to liquefy on the ultrasound imaging window. The GUI identified hexagonally-packed points with 0.5 mm separation within the planned location. At each location, 200 pulses of 0.66 *μ*s duration (one cycle duration of the 1.5 MHz fundamental frequency) and 35 MPa peak negative pressure were applied. The pulse duration was reduced compared to the clot insonation to increase the precision of liquefaction [18]. Following histotripsy exposure, phantoms were sectioned and photographed with a DSLR camera (Nikon D3400, 24 MP resolution, Minato, Tokyo, Japan) to visualize the resultant liquefaction zone. Gross images of the phantom were converted to grayscale and segmented using Otsu’s method to delineate liquefied and intact regions [34]. The phantom images were co-registered with the pre-treatment planning B-mode images using fiducial markers embedded within the agarose (Fig. 4). For treatment schemes that ablated an area of the phantom, the Dice similarity coefficient was computed to compare the predicted (pre-treatment planning points) and actual areas of phantom liquefaction. The predicted liquefaction area at each insonation location (green dots, Fig. 4A) was estimated based on the focal zone area exposed to a peak negative pressure in excess of 27.4 MPa (elliptical area with major and minor axes of 3.4 mm and 0.5 mm, respectively), the anticipated threshold for bubble cloud generation in agarose gel [19]. The Hausdorff distance *h* was also computed to gauge the maximum distance between the predicted and actual liquefaction locations [35]:

(3) $$h(A,B)=\underset{a\in A}{\text{max}}\left\{\underset{b\in B}{\text{min}}\left\{d(a,b)\right\}\right\}$$

where *A/B* are the predicted/actual areas of ablation, *a*/*b* are spatial locations of predicted/actual ablation, and *d*(*a,b*) is the distance between these points.

## III. RESULTS

### A. Tracking Model Vessel

Studies to gauge the precision of the system in tracking a model vessel provide information regarding repeatability of the image guided, user executed system, and accuracy of the interpolation algorithm to translate the histotripsy focus along the intended treatment path. Three orientations of the model vessel (lumen) were tested: 1. Parallel relative to the bottom of the tank (orientation 1), 2. Angled 10.1° ± 1.0° relative to the first orientation in the range dimension of the imaging plane (see Fig. 2), and 3. Angled 13.1° ± 0.8° and 7.2° ± 1.3° relative to the first orientation in the range and elevational dimensions of the imaging plane (orientation 3), respectively. The first orientation required the cobot to translate along one dimension relative to the transducer (elevational dimension of the imaging plane), and would be considered a best-case scenario for accurate interpolation of positioning. Subsequent orientations of the lumen span the range of angulations expected for a human femoral vein relative to the skin of the leg [36].

**Fig. 5.**
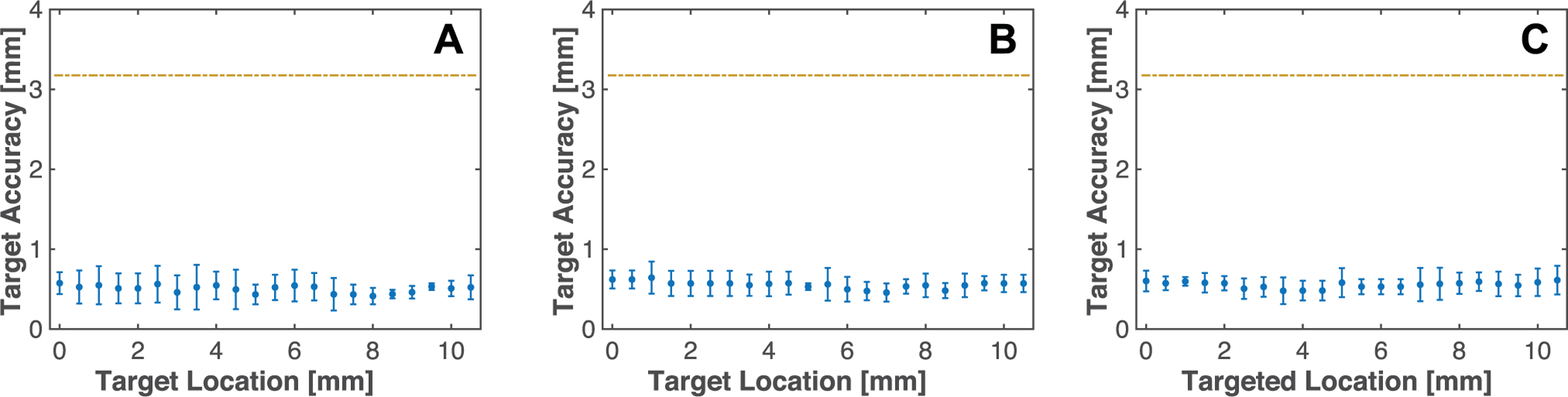
Accuracy of pre-treatment planning reported as the relative distance between the transducer focus and centroid of lumen. The target accuracy is reported at each targeted position of the model vessel. Panel A corresponds to orientation 1 (translation of the source along the elevational dimension of the ultrasound imaging plane), Panel B corresponds to orientation 2 (10.1° ± 1.0° angle relative to the first orientation in the range dimension of the imaging plane), and Panel C corresponds to orientation 3 (13.1° ± 0.8° and 7.2° ± 1.3° relative to the first orientation in the range and elevational dimensions of the imaging plane, respectively). The dashed yellow line corresponds to the inner radius of the lumen. Data points represent the mean and error bars are the standard deviation (n = 5).

For all three orientations of the model vessel, no trends were observed in treatment planning (i.e. placement of the imaging array/transducer relative to the center of the lumen) as a function of position along the model vessel. The average error in placement along all positions along the model vessel were 0.5 ± 0.2 mm, 0.6 ± 0.1 mm, and 0.6 ± 0.2 mm, for each respective orientation (Fig. 5). Thus, the cobot system achieved submillimeter accuracy for tracking all model vessel orientations.

### B. Bubble Cloud Targeting of Clot

**Fig. 6.**
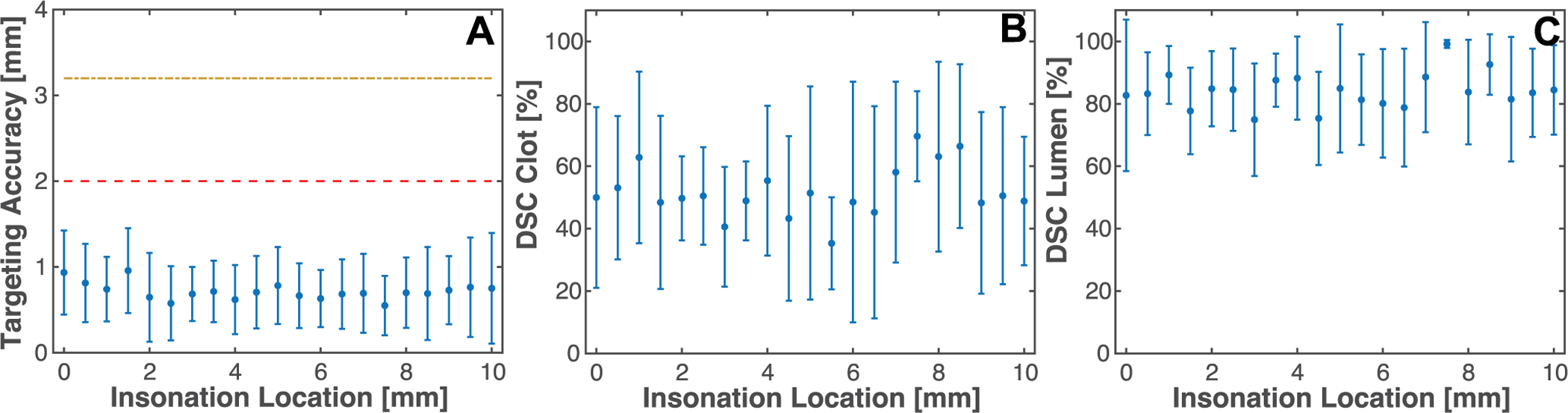
(A) Accuracy of targeted bubble activity within the clot as gauged by the centroid of bubble cloud emissions tracked with passive cavitation imaging and the center of the clot. The dashed red line corresponds to the average outer radius of the clot, and the dashed and dotted yellow line corresponds to the inner radius of the lumen. (B) Dice Similarity Coefficient (DSC) comparing area of cavitation emissions within 1 dB of maximum acoustic power tracked with passive cavitation imaging, and area of the clot cross section function of insonation location along the length of the clot. (C) Dice Similarity Coefficient (DSC) comparing area of cavitation emissions within 1 dB of maximum and area of the lumen cross section as function of insonation location along the length of the clot.

Ten clots from the blood of four individual donors were targeted with the cobot-histotripsy system. The model vessel was approximately parallel with the tank (orientation 1, see Fig. 5) to prevent movement of the clot during histotripsy exposure. On average, the centroid of bubble cloud emissions tracked with passive cavitation imaging was within 0.7 ± 0.4 mm of the center of the clot (approximate clot radius of 2 mm, see Fig. 6A). The position of bubble cloud relative to the clot was independent of the position targeted along the length of the clot, indicating there was no drift of the arm substantially outside the intended treatment path. Dice similar coefficient analysis indicated that 68.8 ± 28.1% of the strongest bubble cloud emissions were contained within the clot, and 91.1 ± 13.6% within the lumen. The clot mass loss, the metric of thrombolytic efficacy, was 18.9 ± 9.3%.

### C. Accuracy of Ablation

**Fig. 7.**
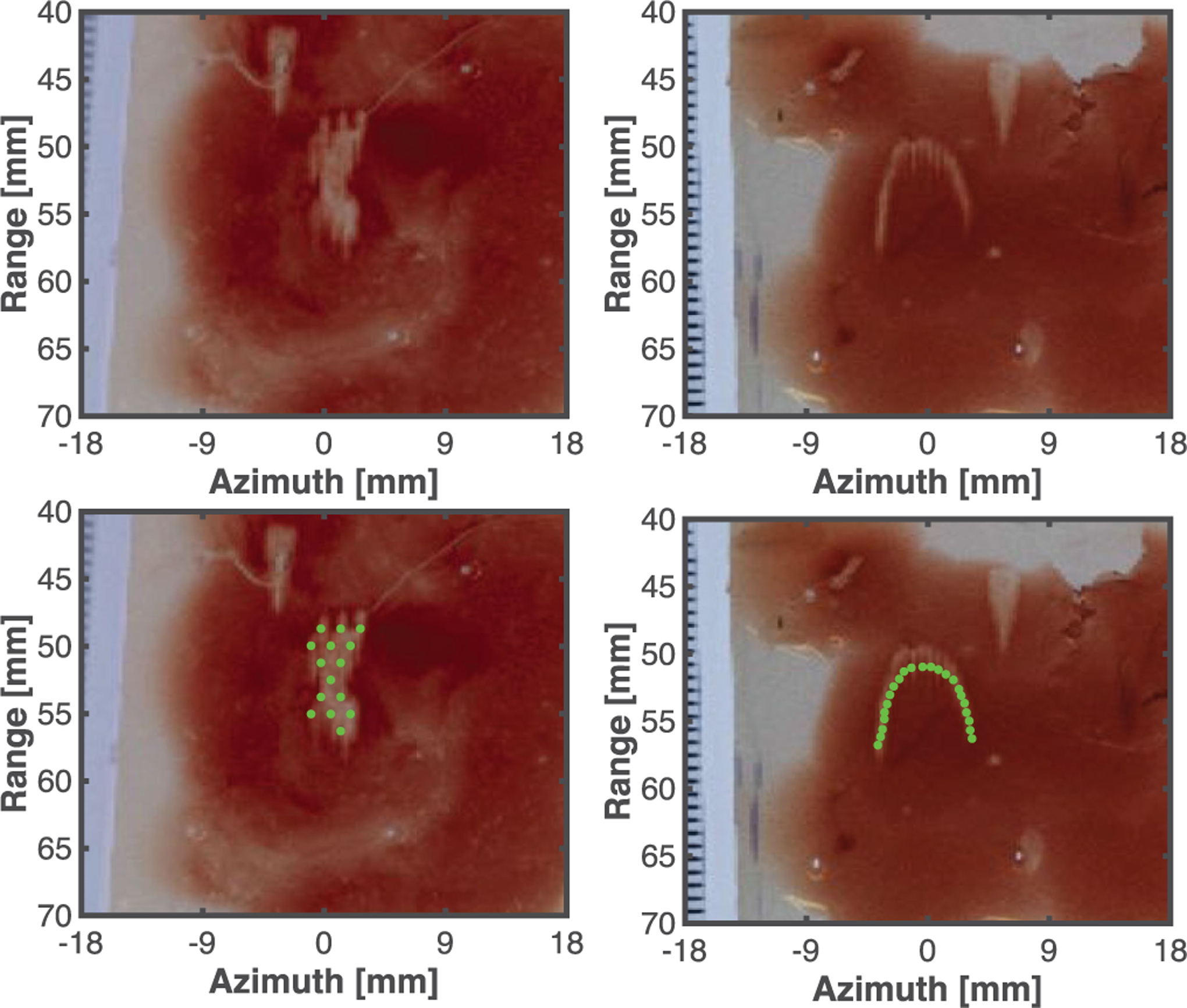
Representative liquefaction zones when an area (Left Column) and path (Right Column) were targeted in the phantom. Locations targeted by the histotripsy source within the phantom are noted by the green dots in the bottom row. Single cycle pulses were used to generate precise ablation zones at pre-defined locations in the phantom.

Histotripsy pulses were applied to 12 red blood cell layers. Good qualitative agreement was observed visually between the predicted liquefaction areas based on pre-treatment planning and the actual liquefaction zone, as indicated in Fig. 7. The ablation zone extended beyond the intended area along the central axis of the histotripsy source (range dimension in Fig. 7), the largest diffractive axis of the source [5]. When areas within the phantom were targeted, the Dice similarity coefficient was 77.8 ± 2.5%. The Hausdorff distance, a metric of the longest distance between the predicted and actual ablation areas, was 1.6 ± 0.6 mm for all targeted regions. The focal zone is elongated along the central axis of the histotripsy source (range dimension in Fig. 7) compared to the other axes, and ablation along this axis had the strongest influence on the reported Hausdorff distance.

## IV. DISCUSSION AND CONCLUSIONS

### A. General Observations

In this study, a cobot system was employed to enable histotripsy ablation over an extended area. Several facets of the cobot provided a significant improvement in transducer positioning relative to the robotic systems used previously for the administration of therapeutic ultrasound [12], [13], [14]. Freedrive mode enabled course placement of the source without the need for input/output communication with the system. The GUI provided a means for fine adjustments of the relative transducer position. The GUI also facilitated precise re-positioning of the source to a given waypoint with high accuracy (0.03 ± 0.01 mm, n = 50 in preliminary study, consistent with the manufacturer specifications). The ability to re-position the source with high precision at waypoints will enable reviewing locations-of-interest set during the pre-treatment planning phase of the therapy after histotripsy exposure.

A limitation of the system is a lack of optical input for visualizing changes in the path due to, for instance, respiratory motion [37]. For the ablation of venous thromboembolism, predominately in the iliofemoral venous segments of the leg, very little respiratory motion is expected [38]. To prevent bulk movement during the application of the histotripsy therapy, the patient leg could be restrained physically. For deep-seated tissues, such as the liver, the target may move over 1 cm during the respiratory cycle [39], and optical tracking scheme or respiratory gating may be required. Ultrasound imaging techniques have been applied previously to compensate for tissue motion [40]. Isoforce programming could be applied to the cobot in order to maintain a constant coupling pressure between the transducer and patient.

### B. Accuracy of Pre-Treatment Planning, Targeting, and Ablation

Tests were conducted on the cobot-histotripsy system to gauge its accuracy pre-treatment planning along a model vessel, the ability to generate bubble activity within an intended target (clot), and the spatial proximity of planned and actual areas of ablation. Submillimeter accuracy was achieved in terms of interpolating the treatment path along the model vessel and generating intense bubble activity along the length of the clot. Similar accuracy has been observed in other studies using cobots in image-guided systems [12], [16]. The average distance between the lumen centroid and histotripsy focus for all three orientations of the model vessel was 0.6 ± 0.2 mm, well within the average size of a human femoral vein [8], [41]. The transducer focus was placed manually within the center of the lumen, which may account for the discrepancy between the targeted and actual positioning of the system. To ensure accurate placement of waypoints along a target path, precise image guidance is critical for cobot-assisted histotripsy therapy.

Based on passive cavitation imaging, the strongest bubble cloud emissions were on average 0.7 mm from the center of the clot, and the majority of acoustic emissions were contained within the clot. Some degree of acoustic energy was noted outside the clot as well. Passive cavitation images are diffraction limited, and the extent of cavitation activity along the range dimension (see Fig. 3) beyond the clot may be overestimated [27], [30]. There may are advantages to the presence of bubble activity outside the extent of the clot. In addition to ablation in the bulk of tissues, histotripsy bubble activity erodes tissue efficiently at a fluid/tissue interface [42]. Further, bubble-induced convective flows in the fluid increase the uptake of lytic drugs within a clot [43]. Therefore, bubble activity in the perfusate outside the clot (but inside the lumen) may be advantageous for histotripsy when combined with a lytic therapy [1], [24]. Over 90% of strong bubble activity was contained within the lumen, indicating minimal off-target effects. However, the system should be evaluated in a relevant animal model to demonstrate accurate targeting of thrombi *in vivo* with minimal collateral damage for confirmation.

Thrombolytic efficacy was reduced for the pulsing scheme used here compared to previous studies, in part due to the reduced total histotripsy exposure time per clot (500 pulses/location in this study vs. 2000 pulses/location in previous studies [1], [24]). Accurate cobot clot targeting was the primary focus of this study, with thrombolytic efficacy as a secondary metric. Future studies will explore the use of the cobot for advanced translational schemes to maximize thrombolytic efficacy [18].

Good correlation was observed between predicted and actual phantom liquefaction zones, as indicated by an average Dice similarity coefficient of 78%, and average Hausdorff distance of 1.6 mm. Ablation accuracies of 0.5 to 4.2 mm have been observed with other robot-assisted focused ultrasound ablation systems [12], [44], [45], within the range observed here. Using a five-axis robotic system to translate a focused source to generate thermal lesions, Tang *et al.* observed nearly 100% ablation of the zone targeted [46]. Overtreatment was also observed, with thermal necrosis extending beyond the targeted region. In contrast, the liquified areas in this study were typically smaller than the predicted liquefaction area based on pre-treatment planning. The discrepancy between the two studies may reflect the variation in ablative mechanisms. Thermal ablation zones diffuse throughout the extent of the focal region due to absorption of the ultrasound energy. In contrast, histotripsy ablation is limited in close proximity to the bubble activity [47]. To ensure ablation of the target, a margin could be added to the region of interest of 1 to 2 mm (the range of Hausdorff distances observed in this study), consistent with the clinical practice for other image-guided therapies [48]. Alternatively, a different histotripsy insonation scheme could be used. A single-cycle excitation was employed for phantom histotripsy exposure to generate the smallest possible liquefaction volume [18]. The means by which histotripsy generates bubble clouds differ with the pulse duration [4]. The extent of the bubble cloud, and therefore the extent of the ablation zone, is proportional to the pulse duration [6]. The correlation between pre-treatment planning and actual ablation may therefore increase with the application of multi-cycle pulses.

There may be other reasons for the discrepancy between the predicted and actual liquefaction areas. The predicted liquefaction area was based on the focal zone dimensions of the transducer. The pulse repetition rate employed in this study is similar to that used in other histotripsy clot ablation studies [1], [14], but may alter the liquefaction zone dimensions due to bubble clouds that persistent between the application of pulses [49]. The overall precision of the system was dependent on the quality of image guidance, here dictated by the ultrasound imaging system. The red blood cell layer is thin compared to the elevational thickness of the L11-5v array (500 *μ*m for the red blood cell layer versus greater than 2 mm elevational thickness for the array), making it difficult to accurately align the imaging plane with the target. As the imaging array is confocal with the histotripsy source, the focal region of the transducer may be slightly angulated relative to the red blood cell layer, and therefore a smaller area of the red blood cell layer may be liquefied relative to the predicted area based on the estimated focal volume. The linear array used in this study is consistent with the arrays employed clinically to assess vascular thrombosis [50]. However, the elevational focus of the imaging array is much shorter than the depth of the focus for the histotripsy source (18 mm versus 60 mm). Methods to improve ultrasound imaging at depth, including chirp-coded excitation [51], [52], may help to improve visualization of histotripsy targets and bubble activity. A fixed number of histotripsy pulses were applied at each location, whereas there is a known threshold cumulative passive cavitation imaging acoustic power associated with histotripsy ablation [33]. Implementation of a feedback scheme for translating the histotripsy source only after this threshold has been reached may improve overall histotripsy treatment efficacy.

### C. Limitations

There are a number of limitations to this study that prevent generalizability of the findings. The use of *in vitro* materials enables fiducial markers to be embedded within the phantom for precise registration of pre-treatment planning and *post hoc* visualization of the liquefaction zone (± 0.5 mm [9]). However, this *in vitro* approach may not be representative of tissue ablation *in vivo*. Further, a two-dimensional ablation area was analyzed for the phantoms, whereas a volume was ablated. Clots were limited to 1 cm in length in this study to facilitate introduction into the model vessel, whereas the burden of venous thromboembolism can be several centimeters in length [7]. The clots did not occlude the entire cross section of the model vessel. Studies to compare the predicted and actual ablation location were conducted in a phantom to enable precise registration of *in situ* imaging with gross visualization of the treatment area. However, the phantom liquefaction profile observed here may not be representative of *in vivo* thrombus ablation. Imaging was conducted primarily through a water acoustic path, which has little to no acoustic attenuation [53]. The femoral vein will be ~2–3 cm below the surface of the skin [8], [41], which will reduce visibility of the clot and therefore targeting. The acoustic emission centroid was a primary metric of bubble activity location in this study, in part due to the limited range resolution of the passive cavitation images [27]. Advanced beamforming techniques are under development to improve the resolution of passive cavitation imaging [54], [55], [56], but have yet to be investigated in the context of predicting histotripsy ablation.

### D. Summary

The treatment of large ablation histotripsy ablation will require translation of the histotripsy source. Here, an image-guided cobot histotripsy system was tested for the accuracy of interpolation, targeting, and ablation. Submillimeter accuracy was obtained in terms of targeting accuracy, and the Hausdorff distance between the targeted location and final ablation zone was less than 2 mm. While tested in the context of thrombus ablation, the system would have utility for targeting any clinical targets for histotripsy.

## References

[1] Bollen V , “In vitro thrombolytic efficacy of single-and five-cycle histotripsy pulses and RT-PA,” Ultrasound Med. Biol, vol. 46, no. 2, pp. 336–349, Feb. 2020. 31785841 10.1016/j.ultrasmedbio.2019.10.009 PMC6930350

[2] Shi A , “Integrated histotripsy and bubble coalescence transducer for thrombolysis,” Ultrasound Med. Biol, vol. 44, no. 12, pp. 2697–2709, Dec. 2018. 30279032 10.1016/j.ultrasmedbio.2018.08.013 PMC6215517

[3] Khokhlova VA , “Histotripsy methods in mechanical disintegration of tissue: Towards clinical applications,” Int. J. Hyperth, vol. 31, no. 2, pp. 145–162, Mar. 2015. 10.3109/02656736.2015.1007538 PMC4448968 25707817

[4] Bader KB, Vlaisavljevich E, and Maxwell AD, “For whom the bubble Grows: Physical principles of bubble nucleation and dynamics in histotripsy ultrasound therapy,” Ultrasound Med. Biol, vol. 45, no. 5. pp. 1056–1080, 2019, doi: 10.1016/j.ultrasmedbio.2018.10.035. 30922619 PMC6524960

[5] Rosnitskiy PB , “Design of HIFU transducers for generating specified nonlinear ultrasound fields,” Ultrason. Ferroelectr. Freq. Control. IEEE Trans, vol. 64, no. 2, pp. 374–390, Jan. 2017. 10.1109/TUFFC.2016.2619913 PMC5300962 27775904

[6] Maxwell AD , “Cavitation clouds created by shock scattering from bubbles during histotripsy,” J. Acoust. Soc. Am, vol. 130, no. 4, p. 1888, 2011. 21973343 10.1121/1.3625239 PMC3206907

[7] Go C , “Single- versus multiple-stage catheter-directed thrombolysis for acute iliofemoral deep venous thrombosis does not have an impact on iliac vein stent length or patency rates,” J. Vascular Surg. Venous Lymphatic Disorder, vol. 7, no. 6, pp. 781–788, 2019, doi: 10.1016/j.jvsv.2019.05.010. PMC7917433 31495769

[8] Hertzberg BS, Kliewer MA, D. A. DM “Sonographic assessment of lower limb vein diameters: Implications for the diagnosis and characterization of deep venous thrombosis,” Am Roentgenol, vol. 168, no. 5, pp. 1253–1257, 5 1997. 10.2214/ajr.168.5.9129422 9129422

[9] Bader KB , “Post Hoc analysis of passive cavitation imaging for classification of histotripsy-induced liquefaction in vitro,” IEEE Trans. Med. Imag, vol. 37, no. 1, pp. 106–115, Jan. 2018. 10.1109/TMI.2017.2735238 PMC5816682 28783627

[10] El Zaatari S , “Cobot programming for collaborative industrial tasks: An overview,” Rob. Auton. Syst, vol. 116, pp. 162–180, Jun. 2019.

[11] Cherubini A , “Collaborative manufacturing with physical human-robot interaction,” Robot. Comput. Integr. Manuf, vol. 40, no. C, pp. 1–13, Aug. 2016.

[12] An CY , “An Ultrasound imaging-guided robotic HIFU ablation experimental system and accuracy evaluations,” Appl. Bionics Biomech, vol. 2017, no. 2, pp. 1–8, 2017. 10.1155/2017/5868695 PMC5406740 28487622

[13] Chauhan S and ter Haar G, “FUSBOTUS: Empirical studies using a surgical robotic system for urological applications,” AIP Conf. Proc, vol. 911, no. 1, pp. 117–121, Jun. 2007.

[14] Zhang X , “Noninvasive thrombolysis using microtripsy: A parameter study,” IEEE Trans. Ultrasonics, Ferroelectr. Freq. Control, vol. 62, no. 12, pp. 2092–2105, Dec. 2015. 10.1109/TUFFC.2015.007268 PMC4824290 26670850

[15] Alnaghy S , “A six-degree-of-freedom robotic motion system for quality assurance of real-time image-guided radiotherapy,” Phys. Med. Biol, vol. 64, no. 10, pp. 105013–105021, 5 2019. 30986773 10.1088/1361-6560/ab1935

[16] Bell MAL and Shubert J, “Photoacoustic-based visual servoing of a needle tip,” Sci. Rep, vol. 8, p. 15519, Oct. 2018. 30341371 10.1038/s41598-018-33931-9 PMC6195562

[17] Maxwell AD , “Noninvasive thrombolysis using pulsed ultrasound cavitation therapy - histotripsy,” Ultrasound Med. Biol, vol. 35, no. 12, pp. 1982–1994, Dec. 2009. 19854563 10.1016/j.ultrasmedbio.2009.07.001 PMC2796469

[18] Zhang X , “Histotripsy thrombolysis on retracted clots,” Ultrasound Med. Biol, vol. 42, no. 8, pp. 1903–1918, Aug. 2016. 27166017 10.1016/j.ultrasmedbio.2016.03.027 PMC4912870

[19] Maxwell AD , “Design of a focused ultrasound transducer for histotripsy-thrombolytic combination therapy,” J. Acoust. Soc. Am, vol. 145, no. 3, p. 1747, Apr. 2019.

[20] Maxwell AD , “A prototype therapy system for transcutaneous application of boiling histotripsy,” Ultrason. Ferroelectr. Freq. Control. IEEE Trans, vol. 64, no. 10, pp. 1542–1557, Oct. 2017. 10.1109/TUFFC.2017.2739649 PMC5871228 28809681

[21] Report of Task Group #13, “Cardiac catherization equipment performance,” AAPM Rep. 070, 5 2001.

[22] Szabo TL, Diagnostic Ultrasound Imaging: Inside Out (Biomedical Engineering), 1st ed., New York, NY, USA; Orlando, FL, USA; San Diego, CA, USA: Academic Press, 2004.

[23] Maxwell AD , “Noninvasive treatment of deep venous thrombosis using pulsed ultrasound cavitation therapy (histotripsy) in a porcine model,” J. Vascular Interv. Radiol, vol. 22, no. 3, pp. 369–377, Mar. 2011. 10.1016/j.jvir.2010.10.007 PMC3053086 21194969

[24] Bader KB , “Efficacy of histotripsy combined with RT-PA in vitro,” Phys. Med. Biol, vol. 61, no. 14, pp. 5253–5274, 2016. 27353199 10.1088/0031-9155/61/14/5253 PMC5563443

[25] Holland CK , “Ultrasound-enhanced tissue plasminogen activator thrombolysis in an in vitro porcine clot model,” Thromb. Res, vol. 121, no. 5, pp. 663–673, Jan. 2008. 17854867 10.1016/j.thromres.2007.07.006 PMC2268623

[26] Bader KB, Gruber MJ, and Holland CK, “Shaken and stirred: Mechanisms of ultrasound-enhanced thrombolysis,” Ultrasound Med. Biol, vol. 41, no. 1, pp. 187–196, 2015. 25438846 10.1016/j.ultrasmedbio.2014.08.018 PMC4258471

[27] Haworth KJ , “Quantitative frequency-domain passive cavitation imaging,” IEEE Trans. Ultrason. Ferroelectr. Freq. Control, vol. 64, no. 1, pp. 177–191, 2017. 27992331 10.1109/TUFFC.2016.2620492 PMC5344809

[28] Jensen CR , “Spatiotemporal monitoring of high-intensity focused ultrasound therapy with passive acoustic mapping,” Radiology, vol. 262, no. 1, pp. 252–261, 2012. 22025731 10.1148/radiol.11110670

[29] Haworth KJ , “Using passive cavitation images to classify high-intensity focused ultrasound lesions,” Ultrasound Med. Biol, vol. 41, no. 9, pp. 2420–2434, Sep. 2015. 26051309 10.1016/j.ultrasmedbio.2015.04.025 PMC4526372

[30] Coviello C , “Passive acoustic mapping utilizing optimal beamforming in ultrasound therapy monitoring,” J. Acoust. Soc. Am, vol. 137, no. 5, pp. 2573–2585, 5 2015. 25994690 10.1121/1.4916694

[31] Crum WR, Camara O, and Hill DLG, “Generalized overlap measures for evaluation and validation in medical image analysis,” IEEE Trans. Med. Imag, vol. 25, no. 11, pp. 1451–1461, Oct. 2006. 10.1109/TMI.2006.880587 17117774

[32] Maxwell AD , “A tissue phantom for visualization and measurement of ultrasound-induced cavitation damage,” Ultrasound Med. Biol, vol. 36, no. 12, pp. 2132–2143, 2010. 21030142 10.1016/j.ultrasmedbio.2010.08.023 PMC2997329

[33] Anthony GJ , “Assessment of histotripsy-induced liquefaction with diagnostic ultrasound and magnetic resonance imaging in vitro and ex vivo,” Phys. Med. Biol, vol. 64, no. 9, pp. 095023, 5 2019. 30921780 10.1088/1361-6560/ab143f PMC6706274

[34] Otsu N, “A Threshold selection method from gray-level histograms,” IEEE Trans. Syst. Man. Cybern, vol. 9, no. 1, pp. 62–66, Jan. 1979.

[35] Huttenlocher DP, Klanderman GA, and Rucklidge WJ, “Comparing images using the Hausdorff distance,” IEEE Trans. Pattern Anal. Mach. Intell, vol. 15, no. 9, pp. 850–863, Sep. 1993, doi: 10.1109/CVPR.1992.223209.

[36] Hughes P, Scott C, and Bodenham A, “Ultrasonography of the femoral vessels in the groin: Implications for vascular access,” Anaesthesia, vol. 55, no. 12, pp. 1198–1202, Dec. 2000. 11121931 10.1046/j.1365-2044.2000.01615-2.x

[37] An CY, Hsu YL, and Tseng CS, “An ultrasound-guided robotic HIFU ablation system with respiration induced displacement and time delay compensation,” J. Med. Biol. Eng, vol. 39, no. 5, pp. 796–805, 2019.

[38] Jäger K, Seifert H, and Bollinger A, “M-mode Echovenography: A new technique for the evaluation of venous wall and valve motion,” Cardiovascular Res, vol. 23, no. 1, pp. 25–30, 1989. 10.1093/cvr/23.1.25 2673513

[39] Davies SC, Hill AL, Holmes RB, Halliwell M, and Jackson PC, “Ultrasound quantitation of respiratory organ motion in the upper abdomen,” Br. J. Radiol, vol. 67, no. 803, pp. 1096–1102, 1994, doi: 10.1259/0007-1285-67-803-1096. 7820402

[40] Pernot M, Tanter M, and Fink M, “3-D real-time motion correction in high-intensity focused ultrasound therapy,” Ultrasound Med. Biol, vol. 30, no. 9, pp. 1239–1249, 2004. 15550328 10.1016/j.ultrasmedbio.2004.07.021

[41] Seyahi N , “Ultrasound imaging findings of femoral veins in patients with renal failure and its impact on vascular access,” Nephrol. Dial. Transplant, vol. 20, no. 9, pp. 1864–1867, Aug. 2005. 15985515 10.1093/ndt/gfh942

[42] Xu Z , “Controlled ultrasound tissue erosion,” Ultrason. Ferroelectr. Freq. Control. IEEE Trans, vol. 51, no. 6, pp. 726–736, 2004. 10.1109/tuffc.2004.1308731 PMC2669757 15244286

[43] Datta S , “Ultrasound-enhanced thrombolysis using definity as a cavitation nucleation agent,” Ultrasound Med. Biol, vol. 34, no. 9, pp. 1421–1433, Sep. 2008. 18378380 10.1016/j.ultrasmedbio.2008.01.016 PMC2945910

[44] Anttinen M , “Feasibility of MRI-guided transurethral ultrasound for lesion-targeted ablation of prostate cancer,” Scand. J. Urol, vol. 53, no. 5, pp. 295–302, 2019. 31556779 10.1080/21681805.2019.1660707

[45] Price KD , “Design and validation of an MR-conditional robot for transcranial focused ultrasound surgery in infants,” Med. Phys, vol. 43, no. 9, pp. 4983–4995, 2016. 27587029 10.1118/1.4955174

[46] Tang T , “A high-precision US-guided robot-assisted HIFU treatment system for breast cancer,” Engineering, vol. 4, no. 5, pp. 702–713, 2018.

[47] Bader KB, “The influence of medium elasticity on the prediction of histotripsy-induced bubble expansion and erythrocyte viability,” Phys. Med. Biol, vol. 63, no. 9, 2018. 10.1088/1361-6560/aab79b PMC5959013 29553049

[48] Burnet NG, Thomas SJ, Burton KE, and Jefferies SJ, “Defining the tumour and target volumes for radiotherapy,” Cancer Imag, vol. 4, no. 2, pp. 153–161, 2004. 10.1102/1470-7330.2004.0054 PMC1434601 18250025

[49] Wang T-Y , “An efficient treatment strategy for histotripsy by removing cavitation memory,” Ultrasound Med. Biol, vol. 38, no. 5, pp. 753–766, 5 2012. 22402025 10.1016/j.ultrasmedbio.2012.01.013 PMC3462164

[50] The American College of Radiology, “Practice parameter for the performance of peripheral venous ultrasound examination.” Jun. 2015. 10.1002/jum.15263 32162338

[51] Shekhar H, Huntzicker S, Awuor I, and Doyley MM, “Chirp-Coded ultraharmonic imaging with a modified clinical intravascular ultrasound system,” Ultrason. Imag, vol. 38, no. 6, pp. 403–419, Aug. 2016. 10.1177/0161734615618639 26634777

[52] Bader KB, Severino P, Hendley SA, Anthony GJ, and Bollen V, “High frame rate imaging to enhance the dissolution of histotripsy-induced bubble clouds,” in Proc. IEEE Ultrasonics Symp, 2019, pp. 1539–1542.

[53] Bader KB , “Effect of frequency-dependent attenuation on predicted histotripsy waveforms in tissue-mimicking phantoms,” Ultrasound Med. Biol, vol. 42, no. 7, pp. 1701–1705, 2016. 27108036 10.1016/j.ultrasmedbio.2016.02.010 PMC4899262

[54] Abadi SH , “Frequency-sum beamforming for passive cavitation imaging,” J. Acoust. Soc. Am, vol. 144, no. 1, pp. 198–209, 2018. 30075672 10.1121/1.5045328 PMC6927771

[55] Lyka E, Coviello CM, Paverd C, Gray MD, and Coussios CC, “Passive acoustic mapping using data-adaptive beamforming based on higher order statistics,” IEEE Trans. Med. Imag, vol. 37, no. 12, pp. 2582–2592, 2018, doi: 10.1109/TMI.2018.2843291. 29994701

[56] Gray MD, Lyka E, and Coussios CC, “Diffraction effects and compensation in passive acoustic mapping,” IEEE Trans. Ultrason. Ferroelectr. Freq. Control, vol. 65, no. 2, pp. 258–268, 2018. 29389657 10.1109/TUFFC.2017.2778509

